# Supplementary material for: Characterizing cell-type spatial relationships across length scales in spatially resolved omics data
Source: Nat Commun. 2025 Jan 3;16:350. doi: 10.1038/s41467-024-55700-1 (PMC11699133; doi:10.1038/s41467-024-55700-1)
Supplement: Supplementary file 3 — Supplementary Software 1 [file 41467_2024_55700_MOESM3_ESM.zip › CRAWDAD/docs/1_simulations.html]

1\_simulations


```
library(crawdad)
library(tidyverse)
```

```
## Warning: package 'ggplot2' was built under R version 4.3.1

## Warning: package 'dplyr' was built under R version 4.3.1

## Warning: package 'stringr' was built under R version 4.3.1

## ── Attaching core tidyverse packages ──────────────────────── tidyverse 2.0.0 ──
## ✔ dplyr     1.1.4     ✔ readr     2.1.4
## ✔ forcats   1.0.0     ✔ stringr   1.5.1
## ✔ ggplot2   3.5.1     ✔ tibble    3.2.1
## ✔ lubridate 1.9.2     ✔ tidyr     1.3.0
## ✔ purrr     1.0.1     
## ── Conflicts ────────────────────────────────────────── tidyverse_conflicts() ──
## ✖ dplyr::filter() masks stats::filter()
## ✖ dplyr::lag()    masks stats::lag()
## ℹ Use the conflicted package (<http://conflicted.r-lib.org/>) to force all conflicts to become errors
```

```
ncores = 7
```

# Load data

Load data.frame of cell positions and labels and convert it to an
`sf` object. This is because CRAWDAD builds upon the
`sf` library in R.

```
data(sim)

## convert to sf
cells <- crawdad:::toSF(pos = sim[,c("x", "y")],
                        cellTypes = sim$celltypes)
```

```
## Warning: 'celltypes' does not have levels. Creating levels from values

## creating `sf` object
```

# Make shuffled background

`CRAWDAD` identifies cell type spatial relationships by
comparing cell type organizational patterns in the real data to a set of
null distributions, which are a datasets that the cell labels have been
shuffled at different scales, or resolutions. We can generate this list
of shuffled datasets with the following code:

```
## generate background
shuffle.list <- crawdad::makeShuffledCells(cells,
                          scales = seq(100, 1000, by=100),
                          perms = 3,
                          ncores = ncores,
                          seed = 1,
                          verbose = TRUE)
```

```
## shuffling permutation 1 using seed 1

## 100 unit scale

## 400 tiles to shuffle...

## shuffling permutation 2 using seed 2

## 100 unit scale

## 441 tiles to shuffle...

## shuffling permutation 3 using seed 3

## 100 unit scale

## 441 tiles to shuffle...

## shuffling permutation 1 using seed 1

## 200 unit scale

## 100 tiles to shuffle...

## shuffling permutation 2 using seed 2

## 200 unit scale

## 121 tiles to shuffle...

## shuffling permutation 3 using seed 3

## 200 unit scale

## 121 tiles to shuffle...

## shuffling permutation 1 using seed 1

## 300 unit scale

## 49 tiles to shuffle...

## shuffling permutation 2 using seed 2

## 300 unit scale

## 49 tiles to shuffle...

## shuffling permutation 3 using seed 3

## 300 unit scale

## 64 tiles to shuffle...

## shuffling permutation 1 using seed 1

## 400 unit scale

## 25 tiles to shuffle...

## shuffling permutation 2 using seed 2

## 400 unit scale

## 36 tiles to shuffle...

## shuffling permutation 3 using seed 3

## 400 unit scale

## 36 tiles to shuffle...

## shuffling permutation 1 using seed 1

## 500 unit scale

## 16 tiles to shuffle...

## shuffling permutation 2 using seed 2

## 500 unit scale

## 25 tiles to shuffle...

## shuffling permutation 3 using seed 3

## 500 unit scale

## 25 tiles to shuffle...

## shuffling permutation 1 using seed 1

## 600 unit scale

## 16 tiles to shuffle...

## shuffling permutation 2 using seed 2

## 600 unit scale

## 16 tiles to shuffle...

## shuffling permutation 3 using seed 3

## 600 unit scale

## 16 tiles to shuffle...

## shuffling permutation 1 using seed 1

## 700 unit scale

## 9 tiles to shuffle...

## shuffling permutation 2 using seed 2

## 700 unit scale

## 16 tiles to shuffle...

## shuffling permutation 3 using seed 3

## 700 unit scale

## 16 tiles to shuffle...

## shuffling permutation 1 using seed 1

## 800 unit scale

## 9 tiles to shuffle...

## shuffling permutation 2 using seed 2

## 800 unit scale

## 9 tiles to shuffle...

## shuffling permutation 3 using seed 3

## 800 unit scale

## 16 tiles to shuffle...

## shuffling permutation 1 using seed 1

## 900 unit scale

## 9 tiles to shuffle...

## shuffling permutation 2 using seed 2

## 900 unit scale

## 9 tiles to shuffle...

## shuffling permutation 3 using seed 3

## 900 unit scale

## 9 tiles to shuffle...

## shuffling permutation 1 using seed 1

## 1000 unit scale

## 4 tiles to shuffle...

## shuffling permutation 2 using seed 2

## 1000 unit scale

## 9 tiles to shuffle...

## shuffling permutation 3 using seed 3

## 1000 unit scale

## 9 tiles to shuffle...

## Time was 0.66 mins
```

# Run pairwise analysis

We can identify trends that describe spatial relationships between
pairwise combinations of cell types in our data. `dist`
refers to the distance at which neighbor cells are defined. In this
example, we assess if the neighbors of each cell type are enriched or
depleted in cells of another given cell type compared to each shuffled
scale of the data.

```
## find trends, passing background as parameter
results <- crawdad::findTrends(cells,
                        neighDist = 50,
                        shuffleList = shuffle.list,
                        ncores = ncores,
                        verbose = TRUE, 
                        returnMeans = FALSE)
```

```
## Evaluating significance for each cell type

## using neighbor distance of 50

## Calculating for pairwise combinations

## A

## B

## C

## D

## Time was 0.21 mins
```

```
## convert results to data.frame
dat <- crawdad::meltResultsList(results, withPerms = T)
```

# Visualize results

```
## calculate the zscore for the multiple-test correction
zsig <- correctZBonferroni(dat)
```

Summary visualization of CRAWDAD’s multi-scale cell-type spatial
relationship analysis.

```
vizColocDotplot(dat, reorder = TRUE, zSigThresh = zsig, zScoreLimit = zsig*2) +
  theme(legend.position='right',
        axis.text.x = element_text(angle = 45, h = 0))
```

```
## this function will be deprecated, use vizRelationships instead

## Scale for x is already present.
## Adding another scale for x, which will replace the existing scale.
```

Visualize specific trends.

```
dat_filter <- dat %>% 
  filter(reference == 'C') %>% 
  filter(neighbor == 'B')
vizTrends(dat_filter, lines = T, withPerms = T, zSigThresh = zsig)
```

```
dat_filter <- dat %>% 
  filter(reference == 'A') %>% 
  filter(neighbor == 'B')
vizTrends(dat_filter, lines = T, withPerms = T, zSigThresh = zsig)
```
